# New Insights into Potential Anti-Aging and Fatigue Effects of a Dietary Supplement from the Resveratrol Beverage in Aged SAMP8 Mice

**DOI:** 10.3390/antiox14111337

**Published:** 2025-11-06

**Authors:** Yu-Chien Chen, Ming-Yu Chou, Po-Hsien Li, Ying-Shen Lin, Mei-Due Yang, Ching-Hsin Chi, Ping-Hsiu Huang, Yun-Jhen Wei, Ming-Fu Wang, Chun-Yen Kuo

**Affiliations:** 1 Department of Food and Nutrition, Providence University, Taichung 433303, Taiwanpohsien0105@pu.edu.tw (P.-H.L.);; 2 International Aging Industry Research & Development Center (AIC), Providence University, Taichung 433303, Taiwan; 3 Ph.D. Program in Health and Social Welfare for Indigenous Peoples, Providence University, Taichung 433303, Taiwan; 4 Department of Surgery Department of Clinical Nutrition, China Medical University Hospital, Taichung 40201, Taiwan; 5 Department of Food Science, National Chiayi University, Chiayi City 600355, Taiwan

**Keywords:** resveratrol, passive avoidance task, active shuttle avoidance tests, glycogen

## Abstract

This study investigated the anti-fatigue and anti-aging benefits of continuous intake of resveratrol (RES)-rich beverages. Locomotion and forelimb grip strength performance were significantly improved in medium- and high-dose RES groups. In terms of aging indices, the scores for the medium- and high-dose groups were significantly lower than those of the control group. In the PAT and active shuttle avoidance tests, the three RES groups performed better than the control group. A significant increase in SOD and catalase activity in the liver and a reduction in TBARS and 8-OHdG levels in the brain were observed in the medium- and high-dose groups. Thus, supplementation with RES-rich beverages for 13 weeks significantly improved fatigue, locomotor performance, learning and memory abilities, and liver antioxidant activity and reduced brain peroxide levels in SAMP8 mice.

## 1. Introduction

Aging is marked by a decline in organismal function and a series of notable hallmarks, including genetic and epigenetic changes [1]. The 2021 global life expectancy decreased from 72.8 years in 2019 to 71.0 years due to the Coronavirus disease (COVID-19) pandemic. Furthermore, the global population aged ≥ 65 years is increasing in number and as a percentage of the total population and is expected to rise from 10% in 2022 to 16% in 2050 [2]. In most countries, the health and social welfare of the elderly have long been given low priority in public health policy. Owing to the existence of chronic diseases that are heavy consumers of healthcare services, demographic aging is a global issue that should be actively addressed to reduce the burden of healthcare on society and ensure a high-quality and dignified retirement life for the older adults. Therefore, early diagnosis and intervention are essential to narrow the treatment gap [3].

Resveratrol (RES) (3,4′,5-trihydroxy-stilbene) is a natural phytoalexin, phytoestrogen, and polyphenol found in a broad range of food sources, such as fruits (e.g., red grapes and berries) [4,5,6], vegetables, chocolate, peanuts, and wine. It has numerous biological activities, such as antioxidant, age-delaying, anti-inflammatory, anti-apoptotic, modulating brain function, and learning, anxiety, depression, autism spectrum disorders, schizophrenia, and memory-related behavioral factor improvement [5,7,8,9,10,11,12]. It facilitates neuroprotection, plays an essential anti-inflammatory role in the brain [13], and prevents or improves intestinal inflammation [7].

In vivo studies have demonstrated the effectiveness of RES and have shown that these metabolites are converted to RES in the liver [14]. Lettieri Barbato, D.; Tatulli, G.; Aquilano, K.Ciriolo, M.R. [15] showed that RES could act as a potent enhancer of the ATGL/FA/PPARα pathway, thereby providing an effective natural tool against aging-related metabolic disorders and the onset of inflammatory states. Although orally administered RES is eventually absorbed by intestinal cells through the digestive system [16], the dietary intake of such compounds reaches only a small percentage of the blood and body tissues [17]. Walle, T. [18] reported that humans absorb at least 75% of RES through oral administration. A previous study found that the daily intake of resveratrol from common foods was significantly low (0.783 mg), compared to the beneficial dose of 30–150 mg; therefore, it has been recommended to enhance the bioavailability of resveratrol by developing related supplements and functional drinks [19].

The successful in vivo application of RES presents significant challenges. This study aimed to overcome this challenge by evaluating the anti-aging and anti-fatigue effects of RES-rich beverages in 3-month-old male senescence-accelerated P8 (SAMP8) mice. SAMP8 mice serve as an excellent model for investigating the mechanisms underlying age-related cognitive dysfunction [20]. They naturally develop learning and memory deficits characteristic of aging, with spatial learning impairments detectable as early as 3 months of age, along with a reduced number of neurons and decreased neurogenesis in the hippocampus [21,22]. RES-rich beverages were orally administered to SAMP8 mice using a syringe and needle for 13 weeks at 3.08 (low-dose group), 6.15 (medium-dose group), and 12.3 mL/kg/day (high-dose group). Aging, locomotor performance, fatigue improvement, brain lipid peroxidation (TBARS) content, and learning memory were then evaluated.

## 2. Materials and Methods

### 2.1. Materials

Commercially available beverage samples (15 mL of each bottle) were provided by Shab Ben Fu Ye International Co. (Taichung, Taiwan), the composition as follows: 99.0% water, 0.45% isomalto-oligosaccharide syrup, 0.24% combined fruit juice concentrate (pineapple, orange, passion fruit, banana, orange, mango, papaya, lemon, lime, apple, and white grape), 0.02% grape skin extract, 0.02% longan honey, 0.02% euphorbia extract, 0.02% gum Arabic, 0.01% sugar cane extract, 0.01% rooibos extract, 0.01% vitamin C, 0.01% *Streptococcus epidermidis* ferment, 0.01% lingonberry extract, 0.01% sesame extract, 0.01% corn gum, 0.01% vitamin E, 0.01% sucralose, 0.01% pine bark extract, 0.01% red algae extract, 0.01% maltodextrin, 0.01% silica, 0.01% lecithin, 0.01% L-anti-ascorbyl palmitate, 0.01% rosemary extract, 0.01% artichoke extract, 0.01% corn extract, 0.01% golden silk bird’s nest extract. Resveratrol (250 mg; Merck KGaA, Darmstadt, Germany) was added to the beverages. The nutritional information of the RES-rich beverages was as follows: carbohydrates, 4.03 g; protein, 3.17 g; sodium, 15.22 mg; and energy, 28 kcal. All chemicals were purchased from Sigma-Aldrich^®^ (Merck KGaA, Darmstadt, Germany) as analytical-grade reagents configured for use as received without any reaction unless described otherwise.

### 2.2. Experimental Animals

In this study, 3-month-old male SAMP8 mice, which are appropriate for aging and reproductive function studies, were purchased from the National Laboratory Animal Center (Taipei, Taiwan). All SAMP8 mice were housed in a 30 (W) × 20 (D) × 10 (H) cm^3^ transparent plastic cage placed in an automatically controlled, dust-free room at a temperature of 22 ± 2 °C and relative humidity of 65 ± 5%. An automatic timer managed the light cycle from 07:00 a.m. to 07:00 p.m. under light and 7:00 p.m. to 7:00 a.m. in darkness. The animals were fed ad libitum with AIN-93M standard purified feed and water, which were changed every other day. The experimental animal protocol was approved by the Animal Research Committee of the University of Providence under the IACUC Approval No. 20201218A007. All operation procedures followed the standards outlined in the Committee for the Control and Supervision of Animal Experiments and the National Institutes of Health Guide for the Care and Use of Laboratory Animals.

### 2.3. Animal Experiment Design

The 32 SAMP8 mice were randomly divided into control and experimental groups (0.5-, 1-, and 2-fold doses) (*n* = 8). Each group was orally administered the test sample through the esophagus once a day, and their diet and body weight were recorded throughout the trial.

The administered RES dose was as follows:

- In the low-dose group (0.5-fold), the recommended intake for adults was 0.25 mL/kg BW/day, which is equal to 0.25 mL/kg BW/day × 12.3 = 3.08 mL/kg BW/day for mice.
- In the medium-dose group (1-fold), the recommended intake for adults was 0.5 mL/kg BW/day, which is equal to 0.5 mL/kg BW/day × 12.3 = 6.15 mL/kg BW/day for mice.
- In the high-dose group (2-fold), the recommended intake for adults was 1.0 mL/kg BW/day, which is equal to 1.0 mL/kg BW/day × 12.3 = 12.3 mL/kg BW/day.

**Figure 1:**
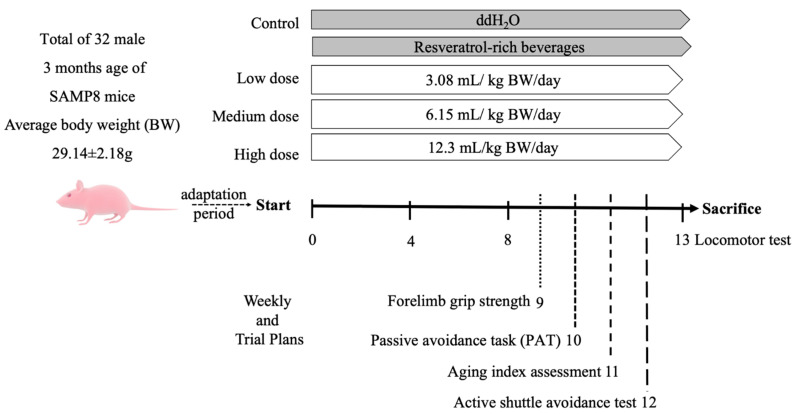
Schedule of animal experiments. Random subgroup adaptation for 7 days, followed by treatment of mice with RES-rich beverages or water for 13 consecutive weeks.

The above doses were calculated based on the actual body weights of the mice for daily intake. The control group was fed sterile water in the same amount as that of the medium-dose group. The trial was performed for 13 consecutive weeks (Figure 1), and the locomotor abilities of the mice were evaluated at weeks 0, 4, 8, and 13. In the 9th week, forelimb grip strength was assessed. Between the 10th and 12th weeks, the aging index, learning, and memory were evaluated using active and single passive avoidance tests, respectively. At the end of the experiment, the mice were sacrificed by cervical dislocation, and blood was collected from their heart. Brain and liver tissues were obtained, weighed, and stored in a −80 °C freezer for later analysis.

### 2.4. Blood Biochemical Analysis

The collected blood was immediately centrifuged at 4 °C and 5000× *g* for 10 min on a microfuge (22R; Beckman Coulter Inc., Brea, CA, USA). Serum analysis of biochemical parameters, including glucose, total protein, albumin, and triglycerides, was performed using a Synchron LX-20 system (Beckman Coulter Inc., Brea, CA, USA) according to the manufacturer’s instructions. Triglyceride, total cholesterol, high-density lipoprotein cholesterol (HDL-C), low-density lipoprotein cholesterol (LDL-C), aspartate transaminase (AST), alanine transaminase (ALT), blood urea nitrogen (BUN), creatinine, creatine kinase (CK), lactate, and uric acid (UA) levels were also examined.

### 2.5. Hepatic Glucose Analysis

The livers of the mice were removed and split immediately after sacrifice, washed with saline, dried with filter paper, and homogenized for hepatic glycogen analysis using a glucose colorimetric assay kit (GOD-POD Method, NZYTech, Lda., Lisboa, Portugal). The assay was performed according to the manufacturer’s protocol.

### 2.6. Locomotor Ability Assessment

**Figure 2:**
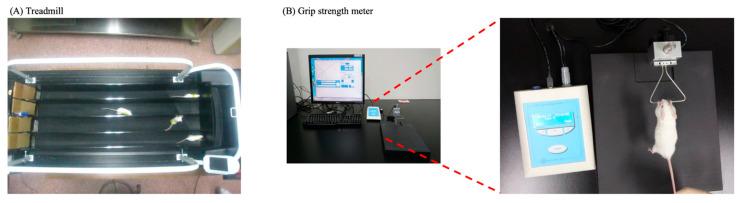
Equipment used in the trial process: (**A**) Treadmill and (**B**) Grip strength meter.

All mice were subjected to locomotion assays on a treadmill (model T306; Diagnostic and Research Instruments Co., Taoyuan, Taiwan) at weeks 0, 4, 8, and 13. The treadmill was equipped with a metal ring and a conductor at the end, and an electric shock (current intensity of 0.6 mA per shock for less than) serves as an electrical stimulus for the mice to stop resting (Figure 2A) and to continue running. The number of shocks (also known as retention frequencies) was recorded subsequently. The test was performed by setting the starting exercise speed to 15 m/min for 20 min, followed by 90 s of rest, 20 min at 20 m/min, followed by 90 s of rest, and finally, 20 min at 25 m/min. The number of electric shocks at different speeds was recorded for each mouse during the trial and used to assess locomotor performance.

### 2.7. Forelimb Grip Strength Assessment

Forelimb grip strength was assessed 30 min after feeding at week 9 using a grip strength meter (Ugo Basile Grip Strength Meter, Cat. No. 47200) (Figure 2B). The mice were positioned to grip the platform with a force sensor grip bar in front of their heads, and the height of the grip bar was adjustable. The tail was grabbed at 1/3 of the root to prompt the mouse to grasp the measuring device, and then it was pulled parallel to the opposite direction so that the body of the mouse and the sensor were in the horizontal plane. The tail of the mouse was pulled rearward until the mouse lost 3 its grip. This process was repeated thrice, and the maximum value was obtained. The underlying principle is that when the animal’s tail is dragged, it naturally grasps the grasping bar in front of it to avoid an involuntary backward movement. The operator’s pulling force exceeded the maximum grasping force. When the animal released the grab bar, the instrument automatically recorded the maximum force.

### 2.8. Aging Index Assessment

The aging index included (1) behavior as an observation of the exploratory reactivity of mice within 30 s and the avoidance passivity of mice while gently pinching the skin on the back of the neck; (2) appearance by observation of hair glossiness, coarseness, loss, and ulcer; eyes: inspection of mucositis around the eyes or edema of the eyelids (peri-ophthalmic lesion); and (3) checking and palpation of the spine lord kyphosis. Each item was rated on a scale of 1–5 according to the definition of each grade. Higher scores indicate more severe aging.

### 2.9. Learning and Memory Trial

#### 2.9.1. Passive Avoidance Task (PAT)

Avoidance memory retention was measured using the protocol described by Labban, S.; Alghamdi, B.S. [12], with minor modifications. The PAT trial was carried out in a 35 (W) × 17 (D) × 20 (H) cm^3^ aluminum chamber (Model E10-15, Coulbourn Instruments, MA, USA) divided into light and dark rooms by a 7.5 (W) × 6.5 (D) cm^2^ guillotine door in the center to separate the mice and allow them to communicate with each other. The metal rods were arranged 1 cm apart in parallel at the bottom of the rooms to provide electricity. The specific operation of the trial was as follows: the mice were initially placed in a light room. After 10 s of environmental adaptation, the guillotine door between the two rooms was opened to allow the mice to freely explore the new environment. Given the dark nocturnal behavior of mice, the guillotine door was quickly closed after the mice entered the dark room. The mice received a shock of 0.5 microamperes for 0.5 s for three consecutive times at a 5 s interval to complete the learning process. Memory was evaluated at 24, 48, and 72 h post-learning by conducting the same operation but without electric shocks. The period of stay in the bright room was recorded, and the maximum duration was 180 s for each trial. The longer their stay in the light room, the better the memory performance of the mice.

#### 2.9.2. Active Shuttle Avoidance Test

The behavior of SAMP8 mice was measured according to the method described by Macheda, T.; Snider, H.C.; Watson, J.B.; Roberts, K.N.Bachstetter, A.D. [23] and Mowrer, O.H. [24] with appropriate modifications using a 35 (W) × 17 (D) × 20 (H) cm^3^ aluminum cage (shuttle cage, Coulbourn instruments Model E10-15) divided into two rooms by a 7.5 (W) × 6.5 (D) cm^2^ guillotine door to separate the mice and allow them to communicate with each other. At the bottom of the cage, metal rods were arranged parallel to each other at 1 cm intervals, and the current was applied during the test. All procedures were controlled using computer programs for the time, sound, light, and electric shock. The mice were initially placed in one of the rooms and given an intertrial interval of 10 s for adaptation, followed by a 10 s conditioned stimulus (CS) of light and sound. If the mouse remained on the same side without any response under the CS system, it was immediately given an unconditioned stimulus (UCS) of 0.3 mA for 5 s; otherwise, no shock was given if the mouse entered the other side of the CS system. The computer determined whether the mice were in the CS or UCS state based on their responses. Each mouse was returned to its cage after undergoing five CS and UCS tests. The same procedure was performed four times at 15–20 min intervals for each of the four consecutive days. The number of avoidance responses of mice under the CS system was recorded to evaluate the effect of RES feeding on the learning and memory of each group of mice.

### 2.10. Determination of Biological Activity Indicators Related to the Aging of Brain Tissues

#### 2.10.1. Quantitative Analysis of TBARS

Quantitative analysis of TBARS was performed as described by Marcus, D.L.; Thomas, C.; Rodriguez, C.; Simberkoff, K.; Tsai, J.S.; Strafaci, J.A.Freedman, M.L. [25], with modifications. Unsaturated fatty acids in cell membranes are attacked by free radicals to produce malondialdehyde (MDA), a lipid peroxidase. MDA forms a red complex with thiobarbituric acid (TBA) at high acidic temperatures (80–100 °C), and this complex has strong absorbance at a wavelength of 550 nm and can be used as an indicator of lipid peroxidation. The brain tissues of mice were placed on ice, diluted with 20-fold 50 mM phosphate buffer (pH 7.0), and homogenized using a homogenizer (Brinkmann Polytron Pt-3000, Kinematica AG, Switzerland) at 100× *g* to obtain a homogenized brain tissue solution.

#### 2.10.2. 8-OHdG Content of Genomic DNA

Brain genomic DNA was extracted in accordance with the standardized protocols for the ReliaPrep^™^ gDNA tissue extraction miniprep system (Promega Co., Madison, WI, USA). A highly sensitive the 8-OHdG check ELISA kit (KOG-HS10E, Japan Institute for the Control of Aging, Nikken SEIL Co., Ltd., Fukuroi, Japan) was used to detect the 8-OHdG concentration. Following the manufacturer’s instructions, 50 μL each of the sample, standard solution, and primary antibody solution was added to a 96-well microtiter plate and incubated overnight at 4 °C. The solution was then removed, washed three times with 250 μL of washing solution, and 100 μL of secondary antibody solution was added and incubated at room temperature for 1 h. The liquid was removed, and the samples were washed thrice with 250 μL of the washing solution. In a protected environment, 100 μL of reconstitution enzyme substrate was added and allowed to react for 15 min until the color changed from dark to light blue. Finally, 100 μL of the reaction-terminating solution was added under protected conditions and allowed to react until the color turned bright yellow. Absorbance was measured at 450 nm. The concentration of 8-OHdG was calculated using a standard curve.

### 2.11. Determination of Antioxidant Biochemical Indicators in the Liver

#### 2.11.1. Measurement of Superoxide Dismutase (SOD) Activity

SOD (Ransod) and control assay kits (SD125 and SD126, Randox Laboratories Ltd., Antrim, UK) were used according to the manufacturer’s instructions. Absorbance at 340 nm can be detected from the reaction of xanthine oxidase with 2-(4-iodophenyl)-3-(4-nitrophenol)-5-phenyltetrazolium chloride (INT) to produce superoxide anion (O^2−^), which is stained red with a formazan red dye. SOD activity (U/mL) was calculated using a standard curve generated from the data. The liver tissue in liquid was mixed with 400 μL of cooled deionized water and stored at 4 °C for 15 min, followed by low-temperature centrifugation (Hermle Z383K). Subsequently, 10 μL of the supernatant and 490 μL of sample diluent were mixed and placed in an automated biochemical analyzer (COBAS MIRA Plus, Roche, Switzerland).

#### 2.11.2. Catalase Activity Measurement

The catalase activity of the samples was determined using the method described by Dhaliwal, J.; Dhaliwal, N.; Akhtar, A.; Kuhad, A.Chopra, K. [26] with some modifications. The underlying principle is that H_2_O_2_ decreases the absorbance of catalase at 240 nm. The livers were collected from the sacrificed mice, cut into 0.15 g sections, added with 250 μL of sodium phosphate buffer (50 mM, pH 7.4), and homogenized in an ice bath with a homogenizer at 100× *g* for 30 s. Liver tissue solutions were prepared and homogenized by adding 40–56 μL of sodium phosphate buffer (50 mM, pH 7.4) and 96 μL of 2% Triton X-100, and then centrifuged at 10,000× *g* for 5 min. For liver homogenates, 10 μL of the supernatant was diluted with sodium phosphate buffer (50 mM, pH 7.0), followed by the addition of 1 mL of 30 mM H_2_O_2_ and uniform mixing. The absorbance values were immediately measured at 240 nm using a spectrophotometer every 15 s, and all changes within 1 min were counted. The change in absorbance per unit time indicates catalase activity. Thus, each unit of catalase was defined as the number of millimoles of H_2_O_2_ consumed per minute.

### 2.12. Statistical Analysis

All data were statistically analyzed using the SPSS statistical package (version 22, IBM Corp., Armonk, NY, USA), and all values are expressed as mean ± standard error (SEM). One-way analysis variance (ANOVA) was used to determine the differences between groups, and Duncan’s multiple range test was used to compare these differences. Statistical significance was set at *p* < 0.05.

## 3. Results and Discussion

### 3.1. Body Weight, Diet, and Water Intake

Table 1 shows the average body weight, diet, and water intake of SAMP8 mice in each group after consuming the RES-rich beverages for 13 weeks. The results showed no significant differences in body weight between the pre- and post-trial periods in any group. Weight gain, food intake, and water consumption also showed significant differences between the control groups and high-dose groups (*p* < 0.05), and this range is comparable to that of SAMP8 mice of the same age with no dietary intervention reported in another study [27]. A previous study showed that rats administered high doses of RES (3000 mg/kg BW/day) showed adverse effects and increased clinical signs of toxicity, such as reduced final body weight and food consumption, elevated BUN, creatinine, alkaline phosphatase, alanine aminotransferase, total bilirubin, and albumin levels, reduced hemoglobin, hematocrit, and red cell counts, and increased white cell counts [28]. However, the no-observed-adverse-effect was observed at 300 mg/kg BW/day of resveratrol in rats [28]. The dose used in the current study was significantly lower than 300 mg/kg BW/day; thus, no associated toxicity or clinical lesions were found. Several human clinical trials of therapy with RES (5 mg/day) for Alzheimer’s disease (AD) [29] and other uses of RES (75 mg twice daily) [30] have also shown its beneficial effects by consistently improving the memory performance of the elderly population and increasing the functional connectivity of the hippocampus [31].

### 3.2. Forelimb Grip Strength

**Figure 3:**
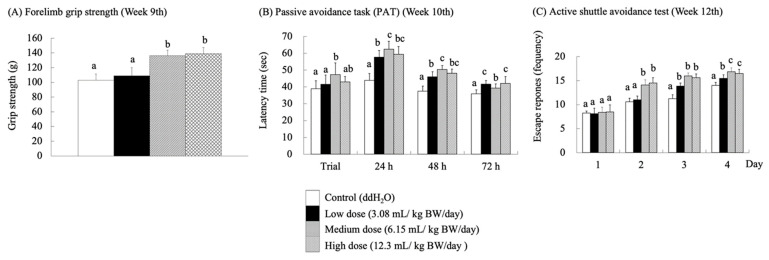
Effects of RES-rich beverage oral administration to SAMP8 mice for 9–12 weeks on forelimb grip strength, passive avoidance task (PAT), and active shuttle avoidance. Values were expressed as mean ± SEM and analyzed by one-way ANOVA. Groups with different letters indicate significant differences (*p* < 0.05).

As the central and peripheral nervous systems age, synaptic degeneration occurs, impairing neurotransmission and leading to cellular atrophy, which inevitably results in cognitive and locomotor dysfunction [32,33,34]. At week 9, a forelimb grip strength test was performed to assess the impact of RES-rich beverages on fatigue in the SAMP8 mice (Figure 3A). The groups receiving medium and high doses showed a significant improvement in forelimb grip performance (*p* < 0.05). Stockinger, J.; Maxwell, N.; Shapiro, D.; deCabo, R.Valdez, G. [35] reported that RES significantly attenuated neuromuscular junction (NMJ) aging in C57BL/6J wild-type mice (24 months) and preserved muscle fiber morphology, thereby accelerating the repair of neurotransmitters. This phenomenon may have contributed to the significant increase in forelimb grip strength in the medium- and high-dose groups of SAMP-8 mice. Nevertheless, Ueta, R.; Sugita, S.; Minegishi, Y.; Shimotoyodome, A.; Ota, N.; Ogiso, N.; Eguchi, T.Yamanashi, Y. [36] observed age-related motor deficits using DOK7 gene therapy and found significant enhancement of NMJ innervation, which led to an increase in motor function and muscle strength in aged mice.

### 3.3. Aging Index

Senescence refers to the general biological processes that occur in organisms as they mature, including decreased efficiency, functional decline, and reduced immune function, ultimately leading to death [37,38]. According to extensive studies at the vertebrate scale, aging is characterized by a decrease in collagen, elastin, bone mineral content, prostatic lesions, and neurogenic diseases [39]. Evidence suggests that some phytochemicals have anti-aging potential, with RES being one of the most studied phytochemicals [38]. The aging index scores are presented in Table 2 for SAMP8 mice fed with RES for 11 weeks. In terms of behavioral aspects, including reactivity and passivity, significantly better performance was observed in the medium-dose (6.15 mL/kg/day) group (*p* < 0.05). Skin assessment, including glossiness, coarseness, hair loss, and ulcers, showed good results in the medium- and high-dose (12.3 mL/kg/day) groups. A significant difference (*p* < 0.05) in skin and hair condition improvement was observed. In particular, the high-dose group showed the best hair loss reduction. The peri-ophthalmic lesion was significantly better in the low-dose group (3.08 mL/kg/day) than in the other groups (*p* < 0.05). No significant difference in lord kyphosis was observed. Overall, the medium- and high-dose groups showed significantly lower aging index scores than the control group, particularly the medium-dose group (*p* < 0.05). This finding indicates that medium- and high-dose RES effectively reduces the degree of aging.

### 3.4. Learning and Memory Skills

Single-PAT and active shuttle avoidance tests were used to evaluate the learning and memory performance of SAMP8 mice fed with the RES-rich beverage. Single PAT was assessed based on the time spent by the mice in the light room. The longer the latency time, the better the learning and memory abilities of mice. An active shuttle-avoidance test was performed to determine the frequency of avoidance responses. In this test, the mice were not subjected to electric shocks for successful avoidance. After the SAMP8 mice were supplemented with RES-rich beverages for 10 weeks (Figure 3B), the latency time in the light room increased significantly (*p* < 0.05) for each experimental group compared to that of the control group at 24 and 48 h. However, the latency duration in the light room decreased in all groups at 72 h. The mice retained their memory because they were given electric shocks on the first day of learning but not at 24, 48, and 72 h. Therefore, the latency in the light room was relatively long at 24 and 48 h. Over time, the ability of the mice to retain memory decreased, leading to a decrease in latency in the light room. In the PAT, RES-rich beverages improved passive avoidance memory in SAMP8 mice. This finding is consistent with previous studies reporting that passive avoidance memory deficits in rat or mouse models of AD were improved by RES treatment [12,40]. Furthermore, Gocmez, S.S.; Şahin, T.D.; Yazir, Y.; Duruksu, G.; Eraldemir, F.C.; Polat, S.Utkan, T. [41] showed that the administration of RES at 20 mg/kg BW/day in DM rats increased their retention scores of PAT to the level of control rats.

The active shuttle avoidance test is a classical test in which mice must combine the presence of a CS with movement between two different rooms to avoid electric shocks [23]. This study combined a standard commercial two-way shuttle case with an automated scoring system that provided quantitative scores with minimal human error. The results were obtained after 12 weeks of RES-rich beverage consumption (Figure 3C). The mice were still in the learning stage on the 1st day of the trial; thus, no significant differences were observed among the groups. On day 2, the escape response frequency in the medium- and high-dose groups was significantly higher than that in the control group (*p* < 0.05). On day 3, the frequency of successful escape responses was significantly higher in all the experimental groups than in the control group (*p* < 0.05). These results showed that the frequency of electric shocks was significantly lower in the medium- and high-dose groups fed with RES-rich beverages than in the control group, indicating that RES enhanced the locomotor performance of SAMP8 mice.

### 3.5. Locomotor Ability

**Figure 4:**
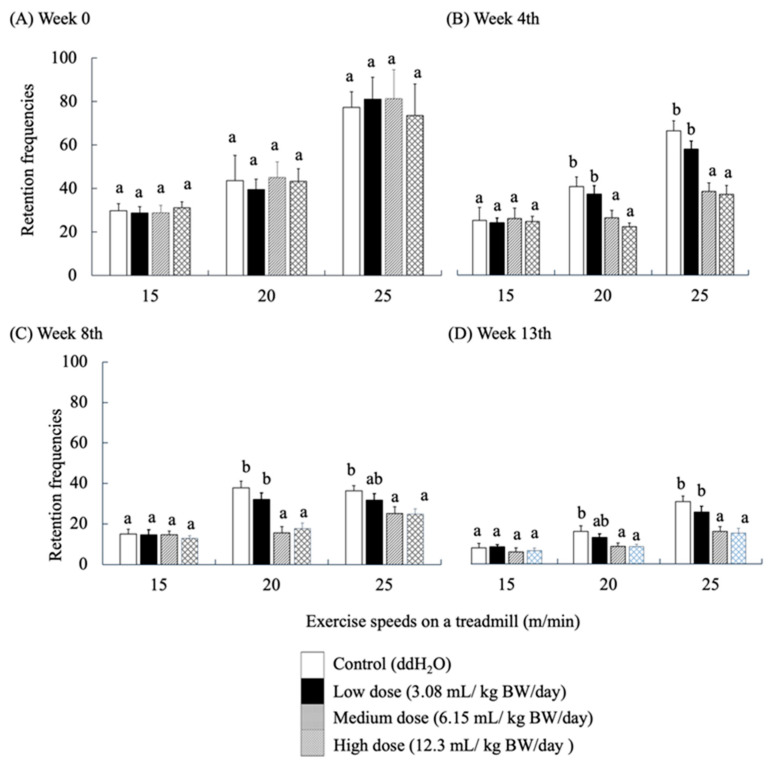
Effects of RES-rich beverage administration to SAMP8 mice for 0, 4, 8, and 13 weeks on locomotor ability assessment. Values were expressed as mean ± SEM and analyzed by one-way ANOVA. Groups with different letters indicate significant differences (*p* < 0.05).

Locomotion performance of mice was evaluated using a treadmill exercise. Retention frequencies (number of electric shocks) were used to evaluate the locomotor ability of the mice as they touched the rear shock area due to fatigue while running. Electric shock was applied as a stimulus to force the mice to continue running. High retention frequency implies a high level of fatigue in mice. The locomotor performance of SAMP8 mice fed with RES-rich beverages at week 0 (Figure 4A) showed that the frequency of shocks increased with each group’s speed and time of locomotion at all three running rates. As the locomotor trial progressed, the frequency of electric shocks showed a significantly decreasing trend in each group of mice (*p* < 0.05).

No significant differences were observed among the groups at this stage of running adaptation. The results of the 4th week also showed no significant difference in the frequency of electric shocks in each group at a running speed of 15 m/min. However, at 20 and 25 m/min, the frequency of electric shocks in the medium- and high-dose groups was significantly lower than that in the control group (*p* < 0.05) (Figure 4B). The same results were observed for the performance of locomotor ability in weeks 8 and 13 (Figure 4C,D). This finding implies that all groups of SAMP8 mice learned to avoid the stimuli. Nevertheless, the response of the medium- and high-dose groups was significantly better than that of the other groups, which is similar to the results described by Oleksiak, C.R.; Ramanathan, K.R.; Miles, O.W.; Perry, S.J.; Maren, S.Moscarello, J.M. [42].

### 3.6. Organ Weight

The weights of the brain, heart, liver, spleen, lung, and kidney were not significantly different in the experimental groups compared to those in the control group after 13 weeks of supplementation with RES-rich beverages in SAMP8 mice (Table 3). The appearance of each organ was observed using the naked eye. No abnormalities, such as swelling or hard masses, were observed, and the color of the organs was normal. Moreover, no SAMP8 mice died during the study. Similarly, dietary supplementation with RES (0.25%) in chronic jet lag-induced C57BL/6 mice showed no obvious changes in the appearance and weight of the liver, kidney, and spleen [43].

### 3.7. Blood Biochemical Analysis

According to the blood biochemical analysis of the mice fed with RES-rich beverages for 13 weeks (Table 4), no significant differences in glucose, total protein, albumin, triglyceride, total cholesterol, lipoprotein cholesterol (HDL-C), low-density lipoprotein cholesterol (LDL-C), aspartate aminotransferase (AST), alanine aminotransferase (ALT), creatine kinase (CK), uric acid (UA), blood urea nitrogen (BUN), and creatinine levels were observed between the groups. Hence, the RES dose used in this study was appropriate and had similar benefits to those used in previous studies [30,31]. A previous study also noted no major changes in the blood biochemical profile, including white blood cells, lymphocytes, monocytes, neutrophils, eosinophils, basophils, red blood cells, packed cell volume, hemoglobin, platelets, mean corpuscular volume, mean corpuscular hemoglobin, and mean corpuscular hemoglobin concentration, following oral administration of various RES doses (15, 30, and 60 mg/kg) in BALB/c mice. However, while significant differences in blood glucose levels were observed in the 15–30 mg/kg treatment group and cholesterol levels in the 15 mg/kg group, all values remained within the normal range [44].

### 3.8. Blood Lactate and Liver Glucose Level

**Figure 5:**
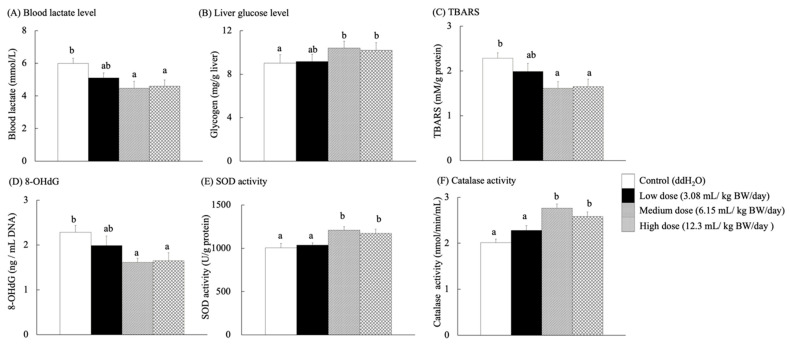
Blood lactate levels, liver glucose levels, and bioactive indexes of aging in the brain and liver tissues for each group of SAMP8 mice after exercise. Different lowercase letters represent significant differences (*p* < 0.05).

Blood lactate level has frequently been used to measure exercise intensity and training performance [45]. Lactate levels in the blood increase under rapidly accelerating ATP energy demands or decreasing oxygen supply, leading to a dramatic reduction in high exercise intensities. Therefore, the mice were fatigued by increasing the treadmill speed to determine their metabolic expenditure and recovery [46]. After the exercise test, the blood lactate concentrations in the medium- and high-dose groups were significantly lower than those in the control group (*p* < 0.05) (Figure 5A). Glycogen is a polymer of glucose stored in the muscles and liver as an important energy deposit [47]. Muscles and liver glycogen play a decisive role in exercise physiology during cycling metabolism, and glycogen as an energy source is an important factor in fatigue [48].

Increased muscle glycogen stores do not necessarily improve exercise performance in GSL30 mice (which overexpress glycogen synthase and can over-accumulate glycogen in the skeletal muscle) [49]. After exercise, the liver glycogen content of SAMP8 mice fed with RES-rich beverages (Figure 5B) did not differ significantly among the groups. Meanwhile, each group showed an increasing tendency for glycogen content in the liver with exercise.

### 3.9. Bioactivity Activity Indicators Related to the Aging of Brain Tissues

Although the brain accounts for only 2% of the total body weight, it consumes 20% of the oxygen in the body and thus requires large amounts of antioxidants to control and prevent oxidation (normal aging) [3]. RES exhibits antioxidant properties by scavenging free radicals and reactive oxygen species (ROS). ROS damage mitochondrial DNA (mtDNA) and cause mutations by accelerating telomere shrinkage, even in non-dividing cells. In addition, ROS are biomarkers of cellular senescence [50,51,52,53]. It upregulates antioxidant enzymes, such as glutathione peroxidase, thereby protecting hippocampal neurons and mitochondria from oxidative stress [11]. The TBARS levels in the brains of SAMP8 mice fed with RES-rich beverages for 13 weeks (Figure 5C) were significantly lower in the medium- and high-dose groups than in the control group (*p* < 0.05). Antioxidant capacity decreases with age [37]. In aged rats, large amounts of protein carbonyls were found, but lipid peroxidation in the heart, liver, kidney, and brain showed no changes [54].

8-OHdG is a biomarker for oxidative DNA damage, linked to cellular aging and functional dysfunction, and may increase cancer risk [55,56]. It has been widely used for its accuracy in identifying and quantifying the mechanisms of leukocyte 8-OHdG, which is essential for understanding its formation, repair, and biological consequences [57]. As shown in Figure 5D, the 8-OHdG content of genomic DNA in the brain was significantly lower in the medium- and high-dose groups than that in the control group (*p* < 0.05). These results confirmed the antioxidant capacity of RES to mitigate oxidative DNA damage because guanine is the most oxidizable of all DNA bases, and 8-OHdG is one of the main oxidation products of any part of the chromosomal DNA [56].

### 3.10. Antioxidant Biochemical Indicators in the Liver

The medium- and high-dose groups showed significantly increased SOD (Figure 5E) and catalase activities (Figure 5F) compared to the control group (*p* < 0.05). A previous study reported that RES (10–40 mg/kg) enhanced SOD activity in D-galactose-induced natural aging and liver injury in Kunming mice by inhibiting the uric acid transport activity of glucose transporter 9 (GLUT9), thereby improving antioxidant and anti-inflammatory status [58]. In contrast, Tiana, L.-Q.; Caib, Q.-Y. [54] reported that aged male Fisher rats showed no change in SOD and glutathione peroxidase levels but exhibited decreased peroxidase activity. They hypothesized that peroxisomes, a single membrane-bound organelle containing antioxidants and oxidative enzymes, may play an important role in the aging process [37,53].

## 4. Conclusions

In this study, supplementation with RES-rich beverages at 6.15 mL/kg/day (medium-dose group) and 12.3 mL/kg/day (high-dose group) was found to be effective in reducing the total score of aging index score in appearance (3.75–4.00), enhancing locomotion performance (forelimb grip strength of 136.18 and 138.91 g; a significantly lower frequency of electric shocks than the control group), relieving fatigue (blood lactate concentration of 4.46 and 4.60 mmol/L), and increasing liver antioxidant enzyme SOD (1209.01 and 1172.55 U/g protein) levels. In the brain, TBARS (1.61 and 1.65 mM/g protein) and 8-OHdG (1.51 and 1.60 ng/mL DNA) levels were also decreased. In terms of learning and memory, single-PAT and active shuttle avoidance tests showed that SAMP8 mice improved their learning and memory abilities. In summary, supplementation with RES-rich beverages might enhance locomotion performance and internal antioxidant capacity and delay the decline of learning memories, thus retarding aging and fatigue. It is important to acknowledge that the beverage formulation included additional phytochemicals, such as grape skin, lingonberry, and pine bark extracts, which may have additive or synergistic effects with RES. Owing to technological and scientific advances, the extraction, characterization, and evaluation of bioactive compounds from food and medicinal plants are now possible. The dietary supplement market for health promotion and disease prevention has a large global commercial market. The potential of RES and its derivatives is of great interest because of their powerful biological activities. Nevertheless, in-depth scientific investigations and full-scale clinical trials are warranted to establish the potential of RES as a new option for managing inflammation in patients with chronic diseases.

## Figures and Tables

**Table 1:** Changes in the body weight, food, and water intake of SAMP8 mice after 13 weeks of administering RES-rich beverages.

Group	Body Weight (g)			Food Intake (g/Day)	Water Consumption (mL/Day)
	Initial	Final	Gain		
Control	29.18 ± 0.33 ^a^	30.05 ± 0.17 ^a^	0.88 ± 0.25 ^a^	5.51 ± 0.07 ^a^	6.07 ± 0.10 ^b^
Low-dose	28.64 ± 1.03 ^a^	29.79 ± 0.92 ^a^	1.16 ± 0.37 ^b^	5.52 ± 0.08 ^a^	5.84 ± 0.12 ^a^
Medium-dose	29.33 ± 0.88 ^a^	30.40 ± 0.67 ^a^	1.07 ± 0.37 ^b^	5.60 ± 0.09 ^a^	5.71 ± 0.11 ^a^
High-dose	29.41 ± 0.80 ^a^	31.05 ± 0.72 ^a^	1.64 ± 0.58 ^c^	5.71 ± 0.06 ^b^	5.99 ± 0.04 ^a^

Values were expressed as mean ± SEM and analyzed by one-way ANOVA. Groups with different letters indicate significant differences (*p* < 0.05).

**Table 2:** Aging index assessment of SAMP8 mice (week 11).

Group	Control	Low-Dose	Medium-Dose	High-Dose
Behavior				
Reactivity	0.75 ± 0.16 ^c^	0.63 ± 0.18 ^b^	0.38 ± 0.18 ^a^	0.38 ± 0.18 ^a^
Passivity	0.63 ± 0.18 ^c^	0.38 ± 0.18 ^a^	0.38 ± 0.18 ^a^	0.50 ± 0.19 ^b^
Skin				
Glossiness	1.25 ± 0.16 ^c^	1.25 ± 0.16 ^c^	0.63 ± 0.18 ^a^	0.75 ± 0.25 ^b^
Coarseness	1.38 ± 0.26 ^d^	1.00 ± 0.27 ^c^	0.50 ± 0.19 ^a^	0.75 ± 0.16 ^b^
Hair loss	0.88 ± 0.30 ^d^	0.75 ± 0.16 ^c^	0.38 ± 0.18 ^b^	0.13 ± 0.13 ^a^
Ulcer	0.25 ± 0.16 ^b^	0.25 ± 0.16 ^b^	0.13 ± 0.13 ^a^	0.13 ± 0.13 ^a^
Eyes				
Periophthalmic lesion	0.75 ± 0.25 ^b^	0.63 ± 0.18 ^a^	0.75 ± 0.25 ^b^	0.75 ± 0.25 ^b^
Spine				
Lordokyphosis	0.63 ± 0.18 ^a^	0.75 ± 0.25 ^b^	0.63 ± 0.26 ^a^	0.63 ± 0.26 ^a^
Total	6.50 ± 0.71 ^a^	5.63 ± 0.89 ^ab^	3.75 ± 0.37 ^b^	4.00 ± 0.42 ^b^

Values were expressed as mean ± SEM and analyzed by one-way ANOVA. Groups with different letters indicate significant differences (*p* < 0.05).

**Table 3:** Comparison of the relative organ weights of SAMP8 mice sacrificed after 13 weeks.

Group	Relative Organ Weights (g/100 g Body Weight)					
	Brain	Heart	Liver	Spleen	Lung	Kidney
Control	1.43 ± 0.10 ^a^	0.65 ± 0.06 ^a^	4.80 ± 0.12 ^a^	0.26 ± 0.02 ^a^	0.66 ± 0.04 ^a^	1.62 ± 0.06 ^a^
Low-dose	1.44 ± 0.04 ^a^	0.62 ± 0.03 ^a^	4.97 ± 0.29 ^a^	0.28 ± 0.03 ^a^	0.68 ± 0.02 ^a^	1.57 ± 0.05 ^a^
Medium-dose	1.47 ± 0.04 ^a^	0.59 ± 0.03 ^a^	4.48 ± 0.17 ^a^	0.23 ± 0.02 ^a^	0.64 ± 0.02 ^a^	1.64 ± 0.06 ^a^
High-dose	1.55 ± 0.07 ^b^	0.62 ± 0.04 ^a^	4.65 ± 0.27 ^a^	0.24 ± 0.02 ^a^	0.65 ± 0.02 ^a^	1.63 ± 0.06 ^a^

Values were expressed as mean ± SEM and analyzed by one-way ANOVA. Groups with different letters indicate significant differences (*p <* 0.05).

**Table 4:** Blood biochemical values of the SAMP8 mice in each group sacrificed after 13 weeks.

Group	A	B	C	D
Glucose (mg/dL)	120.00 ± 6.90 ^a^	113.13 ± 5.02 ^a^	111.75 ± 6.91 ^a^	118.63 ± 8.46 ^a^
Total Protein (g/dL)	5.31 ± 0.18 ^a^	5.39 ± 0.10 ^a^	5.60 ± 0.10 ^a^	5.55 ± 0.12 ^a^
Albumin (g/dL)	3.11 ± 0.09 ^a^	3.18 ± 0.11 ^a^	3.26 ± 0.16 ^a^	3.33 ± 0.15 ^a^
Triglyceride (mg/dL)	107.50 ± 4.74 ^a^	105.75 ± 5.80 ^a^	109.00 ± 4.95 ^a^	103.88 ± 4.47 ^a^
Total Cholesterol (mg/dL)	111.13 ± 4.24 ^a^	114.00 ± 5.07 ^a^	110.25 ± 3.86 ^a^	116.63 ± 4.65 ^a^
HDL (mg/dL)	56.63 ± 4.48 ^a^	54.63 ± 7.02 ^a^	58.38 ± 3.21 ^a^	59.88 ± 5.26 ^a^
LDL (mg/dL)	7.11 ± 0.69 ^a^	7.51 ± 0.74 ^a^	7.10 ± 0.59 ^a^	7.38 ± 0.64 ^a^
AST (U/L)	88.63 ± 3.35 ^a^	87.00 ± 3.85 ^a^	86.25 ± 5.27 ^a^	92.75 ± 2.95 ^a^
ALT (U/L)	65.38 ± 8.60 ^a^	56.75 ± 5.90 ^a^	61.25 ± 6.08 ^a^	58.00 ± 5.84 ^a^
BUN (mg/dL)	29.69 ± 1.65 ^a^	27.34 ± 0.78 ^a^	26.14 ± 1.25 ^a^	25.04 ± 0.91 ^a^
Creatinine (mg/dL)	0.31 ± 0.02 ^a^	0.29 ± 0.02 ^a^	0.33 ± 0.03 ^a^	0.30 ± 0.03 ^a^
Creatine kinase (U/L)	265.75 ± 20.59 ^a^	264.00 ± 19.06 ^a^	266.88 ± 19.37 ^a^	261.38 ± 13.89 ^a^
Uric acid (mg/dL)	4.64 ± 0.46 ^a^	4.34 ± 0.39 ^a^	4.11 ± 0.42 ^a^	4.00 ± 0.52 ^a^

Values were expressed as mean ± SEM and analyzed by one-way ANOVA. Groups with different letters indicate significant differences (*p <* 0.05).

## Data Availability

The data presented in this study are available on request from the corresponding author/Supplementary Materials. The data are not publicly available due to ethical restrictions.

## Supplementary Materials

The following supporting information can be downloaded at: https://www.mdpi.com/article/10.3390/antiox14111337/s1.
